# Environment assisted quantum model for studying RNA-DNA-error correlation created due to the base tautomery

**DOI:** 10.1038/s41598-023-38019-7

**Published:** 2023-07-04

**Authors:** Fatemeh Ghasemi, Arash Tirandaz

**Affiliations:** 1 Department of Energy Engineering, Sharif University of Technology, P.O. Box 11365-9516, Tehran, Iran; 2 Department of Chemistry, Bu-Ali Sina University, Hamedan, Iran

**Keywords:** Biophysics, Biological physics, Quantum physics

## Abstract

The adaptive mutation phenomenon has been drawing the attention of biologists for several decades in evolutionist community. In this study, we propose a quantum mechanical model of adaptive mutation based on the implications of the theory of open quantum systems. We survey a new framework that explain how random point mutations can be stabilized and directed to be adapted with the stresses introduced by the environments according to the microscopic rules dictated by constraints of quantum mechanics. We consider a pair of entangled qubits consist of DNA and mRNA pair, each coupled to a distinct reservoir for analyzing the spreed of entanglement using time-dependent perturbation theory. The reservoirs are physical demonstrations of the cytoplasm and nucleoplasm and surrounding environments of mRNA and DNA, respectively. Our predictions confirm the role of the environmental-assisted quantum progression of adaptive mutations. Computing the concurrence as a measure that determines to what extent the bipartite DNA-mRNA can be correlated through entanglement, is given. Preventing the entanglement loss is crucial for controlling unfavorable point mutations under environmental influences. We explore which physical parameters may affect the preservation of entanglement between DNA and mRNA pair systems, despite the destructive role of interaction with the environments.

## Introduction

**Figure 1:**
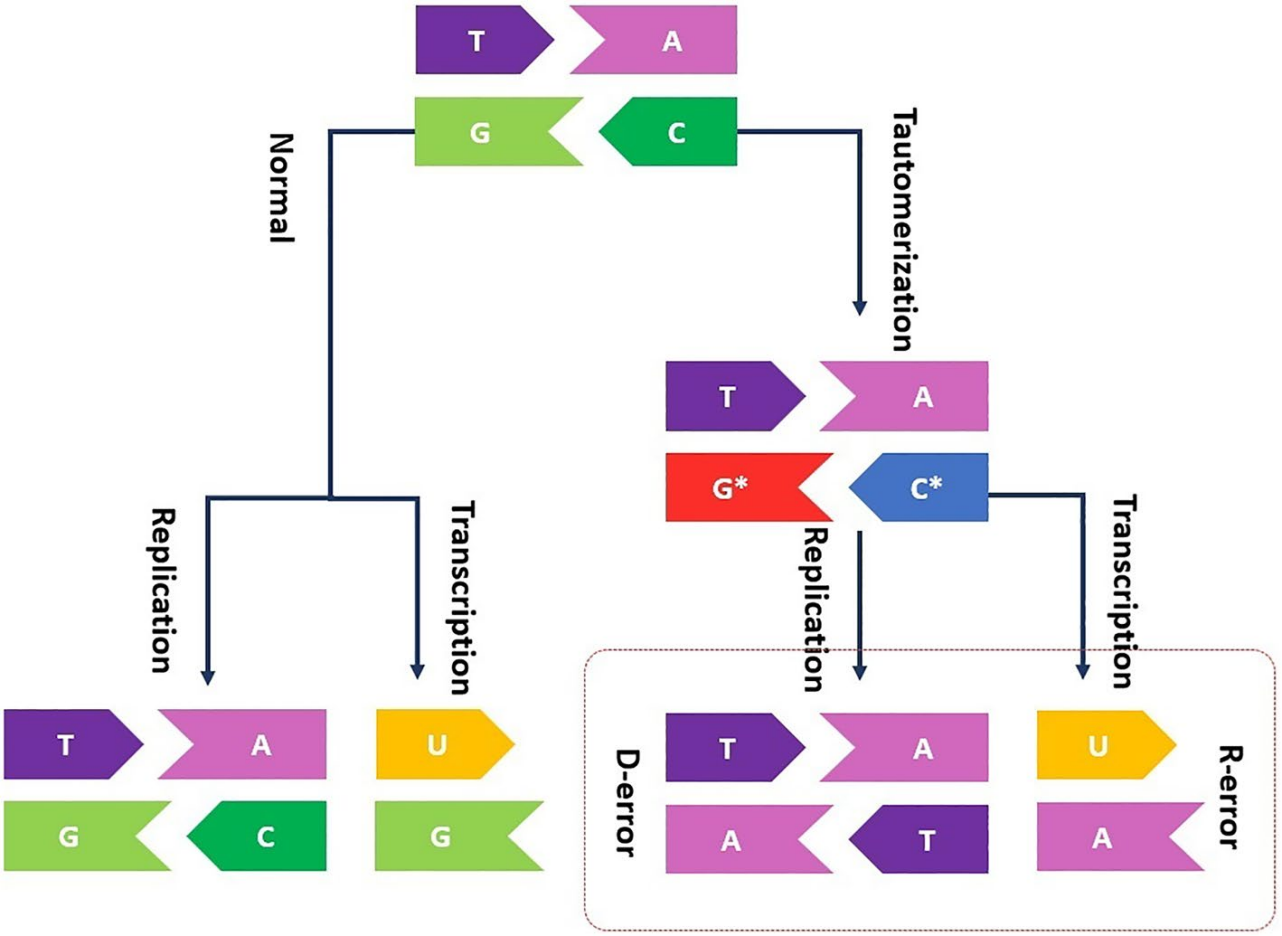
Schematic representation for correlated R-D-error. The left branch shows the normal path of replication and transcription of DNA. This path creates no error in DNA and mRNA. The right branch contains the C-G $\rightarrow$ C*-G* transformation due to proton tunneling in DNA. The replication and transcription of transformed copy of DNA will create DNA and RNA copies, both containing error in the same position, denoted as D-error and R-error, respectively.

As the frontiers of quantum biology predicted, one of the most debating topics in relation to quantum origins of life is the evolution story^1–4^. Thus far, researchers have considered two different Darwinian and Lamarckian mechanisms for evolution process. Darwinian evolution mode occurs at low-stress levels, where random mutations seem to be a dominant source for evolution^5,6^. In contrast, the Lamarckian mechanism happens at high-stress levels, where the adaptive mutations are dominant and environmental factors introduce genomic changes. Here, the mutations target are specific genes and causes of adaptation to the original motive. Through the phenomenon of adaptive or directed mutations individual organisms show suitable plasticity to contribute directly into the evolutionary process by changing their genome. Adaptive mutations are time-dependent and appear only after the cell exposion to a selective substrate^7,8^. For several decades, people have tried to explain how cells can selectively mutate a specific gene in response to environmental signals. Quantum studies of the evolution suggest that adaptive mutations may be generated by environment-induced collapse of the quantum wave function describing DNA in a superposition^9^ of mutated and unmutated states^7,8,10^. Proton tunneling is the way that DNA can become in superposition. Löwdin considering the proton tunneling between two adjacent sites within the H-bonded DNA bases proposed a quantum model for gene mutations^11,12^. The proton tunneling in DNA can cause the transformation like C-G $\rightarrow$ C*-G*. During the replication process, these tautomeric forms can cause incorporation errors in replicated DNA as shown in Fig. 1. If the incorporation errors not become corrected during the proofreading stage it may cause the mutations. For explaining adaptive mutation with the aid of proton tunneling it is necessary the incorporation of error within the coding strand of DNA. The quantum state of this proton can be introduced by a linear superposition of position states for tunneled and not-tunneled proton. Furthermore, an anomalous base-pairing of the tautomeric form can cause the incorporation of an incorrect base into DNA strand during the DNA replication, for instance incorporating base T instead of base C. Subsequent transcription and translation of the mutant form of the gene will result in expression of the mutant phenotype and sitting incorrect amino acid in protein chain^7,8,13^. For describing the adaptive mutation with such a mechanism, the evolving DNA wave function must remain coherent for sufficiently long time to interact with the cell’s environment. The coherence must be maintained during the separation of the two strands of the DNA via helicase^14–18^. There is an intense debate on if both forms of tautomers can exist and dynamically be stable. The strengths of hydrogen bonds within DNA due to the inherently quantum mechanical nature of hydrogen bonding can be affected by nuclear quantum effects^19–21^. Moreover, the unzipping DNA is a complex biological process and involves strong interactions from several proteins^22^. It has been hotly debated for decades if the coherence of tautomers can survive the unzipping helicase^23–25^. McFadden and Al-khalili modeled a specific mutational process involving proton tunneling and investigated the possibility of the coherence to be maintained. They estimate the rate of decoherence for the protons initiating mutational events within DNA using the Zurek model^26^. Accordingly, for a system of mass *m* in a superposition of two position states separated spatially by a distance $\Delta x$ the decoherence time can be defined as $t_D\cong t_R\dfrac{\lambda _T}{\Delta x}$. Where $\lambda _T=\bar{h}\sqrt{2mk_BT}$ denotes the thermal de Broglie wavelength that is temperature, *T*, dependent, and $t_R$ is the relaxation time. Their estimation showed that DNA coding information of tautomeric forms may remain coherent for biologically feasible periods of time^7^. More recently, Slocombe et al. investigations demonstrated that the quantum rate of tautomeric lifetime is significantly higher than the classical rate for a wide range of bath coupling strengths. The proton transfer processes and interconversion between the normal and tautomeric forms occur remarkably quicker than the helicase cleavage timescale^19^. These evidences for surviving coherence between tautomers allowed scientists to hire quantum approaches of evolution on the genome^7^ and cellular level^8^ for describing the various aspects of adaptive mutations^27–30^. Both approaches inspect the situation in which the system under consideration fluctuates between two quantum states labeled as mutated and unmutated states due to the proton tansfer^7,8,11–13^. In the absence of a selective substrate, the mutated and unmutated states are not distinguishable by the environment^7,8^. In such situations the state is said to be stable. The application of the selective substrate destabilizes the fluctuating state that can lead to the generation of the mutant colony. The addition of a specific substrate may result in the collapse of the superposition by rapid decoherence, which corresponds to consecutive monitoring of the state of the system with its environment^31–34^.

Ogryzko argued that the quantum explanation for adaptive mutation can be established with the aid of a particular correlation between ‘R-error’ and ‘D-error’^8,35^. Where the term R-error refers to synthesis of a mutated strand of mRNA due to the recognition of a tautomeric form of a base along the gene by RNA-polymerase. Also, the term D-error specifies a similar mistake made by DNA-polymerase (see Fig. 1). According to him, using a scenario involving both errors is satisfying to describe adaptive mutations. In his model, generating the correlation between R-error and D-error first requires that the RNA-polymerase activity create two superposed branches for the newly transcribed mRNA in the cell due to parental DNA base tautomerization. Furthermore, presence of the substrate is needed to provide enough energy and primary materials for DNA to initiate replication. The DNA-polymerase with a high probability should recognize the same incorrect nucleotide and make exactly the same error as the error made by the RNA-polymerase. Thereby, mutant DNA copies also can be formed, and the colonies of the mutated states may emerge. The possibility of making the same error as the mistake made by the RNA-polymerase for DNA-polymerase guarantees a correlation between the R- and D-errors which can be called the ‘R-D-error correlation.’ Note that both errors must occur simultaneously in a cell. The correlation would have the form $P = {(\text {R}_{\text {er}} ,\text {D}_{\text {er}}) , (\text {R}_{\text {cor}},\text {D}_{\text {cor}})}$ where *P* is the set of possible outcomes consisting of two elements: ($\hbox {R}_{\textrm{er}},\hbox {D}_{\textrm{er}}$), corresponding to combination of R-error and D-error. ($\hbox {R}_{\textrm{cor}},\hbox {D}_{\textrm{cor}}$), refers to combination of no D-error and no R-error.

Taking non-classical correlations between two different parts of the cell into account reveals a prominent feature of quantum mechanics, namely the entanglement^36^. The entangled states are sensitive to the system-environment interaction and can be destroyed quickly due to the environmentally induced decoherence (EID)^10,37^. Here, a related question arises as to what extent this kind of non-classical correlation in the cell is prone to survive from the EID. In different words, how decoherence can play a positive role in stabilizing some non-classical correlations in the macroscopic object of living cell?

After transcription and replication processes within the nucleus and before translation, the daughter DNA and mRNA separate from each other and lie in different places. The daughter DNA remains in the nucleoplasm, and mRNA migrates to the cytoplasm according to their biological tasks. From now on, daughter DNA will be referred to as DNA for simplicity. For such a bipartite system of DNA and mRNA, it is desirable to describe what occurs for the available information of two separated parts after they have correlated for a while. Putting in quantum language, entanglement implies dealing with just a single composite system, instead of two isolated ones. Therefore any change to one subsystem, regardless of the distance between two parts, would influence the other.

In this investigation, we consider a model consisting of the pair of DNA and mRNA located in nucleoplasm and cytoplasm, respectively. Both DNA and mRNA are two-level systems created due to the base tautomerizatio. We use this model to study their correlation and the related entanglement dynamics. The correlation between DNA and mRNA is a primary correlation after the replication and transcription of error containing DNA. Correlation between daughter DNA and amino acids is a secondary correlation and is a consequence of the correlation between mRNA and DNA. We mainly focus on the time-evolution study of the entanglement between DNA and mRNA while they interact with the corresponding environments. We analyze the interaction between DNA and mRNA subsystems and the environments using a framework proposed by Takagi in^38^ for tackling macroscopic quantum tunneling in the presence of the environments. By calculating concurrence of the two entangled qubits, as a measure of entanglement propagation, we comment on the extent of dispersing of entanglement on the whole system. Our approach opens a new perspective to study genetic errors and adaptive mutations through the distinct framework of quantum mechanics^39^.

The paper is organized as follows. In Sect. "Model and Quasi-classical approach", we first describe the model of the entire system consisting of the bipartite mRNA and DNA system and reservoirs, including cytoplasm and nucleoplasm. As we are interested in the interaction between DNA and mRNA subsystems and the environments, in Sect. "Time evolution study of the entangled bipartite system", we examine the dynamics of the bipartite system and solve the related equations in the framework of the perturbation theory. The results are used in Sect. "The concurrence of the bipartite system" to calculate the concurrence of the two entangled subsystems when they are located in different environments. We briefly conclude our results at the very end.

## Model and Quasi-classical approach

Consider a bipartite system $S = S_1 + S_2$ composed of a DNA and mRNA pair denoted by $S_1$ and $S_2$, respectively. Because of tautomerization, both DNA and mRNA can be represented by two-state systems including the un-mutant and mutant forms. To study the error-correlation in DNA and RNA as a result of tautomerization, we consider the time-evolution of the entangled bipartite system coupled to two environments consist of bosonic modes. Hamiltonian of the entire bipartite system and environments can be defined as:

1 $$\begin{aligned} H&=H_S+H_\mathcal {E}+H_{S\mathcal {E}}, \end{aligned}$$

where $H_S=H_{S,1}+H_{S,2}$ and $H_\mathcal {E}=H_{\mathcal {E},1}+H_{\mathcal {E},2}$ are the system and the environment Hamiltonian, respectively. Here, we use $H_{S,1}$ and $H_{S,2}$ to demonstrate the Hamiltonian corresponding to two-state DNA , $S_1$ and two-state mRNA as $S_2$. $H_{\mathcal {E},1}$ and $H_{\mathcal {E},2}$ denote the corresponding Hamiltonian of the harmonic environments coupled to DNA and mRNA, respectively. $H_{S\mathcal {E}}=H_{S_1\mathcal {E}_1}+H_{S_2\mathcal {E}_2}$ represents the interaction Hamiltonian of the DNA and mRNA systems with the nucleoplasm and cytoplasm, respectively which $H_{S_i\mathcal {E}_i}$ has the following form^38^:

2 $$\begin{aligned} H_{{S_{i}{\mathcal {E}_i}}}&= -\sqrt{\dfrac{\tilde{h}}{2}}\sum _{\alpha _i}\omega _{\alpha _{i}}^{3/2}f_{\alpha _i}(\hat{q_i})(\hat{b}_{\alpha _i} +\hat{b}_{\alpha _i}^\dagger )+\dfrac{1}{2}\sum _{\alpha _i}\omega _{\alpha _{i}}^2\lbrace f_{\alpha _i}(\hat{q_i})\rbrace ^2, \end{aligned}$$

where $q_i$ represents the position variable of *i*th system, $\omega _{\alpha _i}$ is the frequency of the harmonic oscillator of the *i*th environment bosonic mode. $\hat{b}_{\alpha _i}^\dagger$ and $\hat{b}_{\alpha _i}$ are the creation and annihilation operators for the oscillators and $f_{\alpha _i}(\hat{q_i})$ describes coupling strength of particle $q_i$ to the $\alpha$th mode of *i*th environmental mode. Here, we use a linearly coupled harmonic environment model, named ’separable interaction’ in which $f_{\alpha _i}(\hat{q_i})=\gamma _{\alpha _i} f(\hat{q_i})$. Where $f(\hat{q_i})$ is an arbitrary function of $q_i$ and $\gamma _{\alpha _i}$ is a positive constant. We set all variables dimensionless, respecting the approach taken by Takagi in^38^, noting that $\tilde{h}$ is also the dimensionless Planck constant which quantifies the extent to which a system is expected to behave as a macroscopic one. Supposing that for a macroscopic two-level system, the potential has the characteristic length $R_0$ with the unit of length and the characteristic energy $U_0$ with the unit of energy, the corresponding characteristic time $\tau _0$ may be introduced as the time required for a particle of mass *M* to pass the distance $R_0$ at a constant speed with the kinetic energy of the order of $U_0$. It is possible to determine $\tau _0$ by the height and the width of the energy barrier. It is usually called the tunneling time. Considering $\tau _0$ as the unit of time, we introduce the parameter $\tilde{h}$, which instead of Planck’s constant appears in a particular dimensionless form of the Schrödinger equation resulting from our choice of units:

3 $$\begin{aligned} \tilde{h}:=\dfrac{\hbar }{U_0\tau _0}=\dfrac{\hbar }{P_0R_0}=\lbrace \dfrac{{\hbar }^2 / M{R_0}^2}{U_0}\rbrace ^{1/2}, \end{aligned}$$

where $P_0$ is the unit of momentum defined as $P_0:=(MU_0)^{1/2}$. The magnitude of $\tilde{h}$ is related to $U_0\tau _0$($=P_0R_0$). It determines how much a near macroscopic system can show quantum traits. In this sense, the condition in which $\tilde{h}\ll 1$ is called the quasi-classical limit. In the region in which $\tilde{h}=1$, the system tends to show its quantumness, purely. If $\tilde{h}$ is too small, it is almost impossible to track its quantum behavior in experience. As a result, the amount of $\tilde{h}$ is a fair measure for quantifying the quantumness of a system on the boundary of being resembled to a classical system. Taking a purely quantum-mechanical approach one prefers to work with $\tilde{h}=1$. If $\tilde{h}$ is too small, it is impossible to detect quantum effects. Then, $\tilde{h}$ quantifies the limit in which quantum mechanical behavior could be expected.

We aim to study the sources of genetic errors and genetic mutations using quantum-mechanical formalism. Thus, we describe the quantum state of the entangled bipartite DNA and mRNA systems which alluded to each other by quantum correlation due to the replication and transcription of tow-state mother DNA strand with $\vert \psi (0) \rangle =\alpha \vert 0\rangle _1\vert 0\rangle _2 +\beta \vert 1\rangle _1\vert 1\rangle _2$. Where similar to the study of a typical two-state system in quantum mechanics, we labeled the excited and ground pure states of the DNA and mRNA as $\vert 1\rangle _i$ and $\vert 0\rangle _i$, respectively. As we specified before $i = 1$ refers to DNA and $i=2$ to mRNA. We denote the states as follows

4a $$\begin{aligned}&\vert 0 \rangle _1 =\dfrac{1}{\sqrt{2}}(\vert \text {D}_\text {cor}\rangle - \vert \text {D}_\text {er}\rangle ) \end{aligned}$$

4b $$\begin{aligned}&\vert 1 \rangle _1=\dfrac{1}{\sqrt{2}}(\vert \text {D}_\text {cor}\rangle + \vert \text {D}_\text {er}\rangle ) \end{aligned}$$

4c $$\begin{aligned}&\vert 0 \rangle _2 =\dfrac{1}{\sqrt{2}}(\vert \text {R}_\text {cor}\rangle - \vert \text {R}_\text {er}\rangle )\end{aligned}$$

4d $$\begin{aligned}&\vert 1 \rangle _2=\dfrac{1}{\sqrt{2}}(\vert \text {R}_\text {cor}\rangle + \vert \text {R}_\text {er}\rangle ). \end{aligned}$$

For the environment $\vert \text {vac}\rangle _1$ and $\vert \text {vac}\rangle _2$ describe initial states of nucloplasm and cytoplasm, respectively. Accordingly, the initial state of the entire system is

5 $$\begin{aligned} \vert \Psi (0) \rangle \rangle&=\vert \psi (0)\rangle \vert \text {vac}\rangle _1\vert \text {vac}\rangle _2 \nonumber \\&=(\alpha \vert 0\rangle _1\vert 0\rangle _2 +\beta \vert 1\rangle _1\vert 1\rangle _2)\vert \text {vac}\rangle _1\vert \text {vac}\rangle _2. \end{aligned}$$

Note that the Hilbert space of the whole $S_1+S_2$ is four-dimensional and this is integrated by all the possible linear combinations of the Kronecker product between the basis elements of Hilbert space of the system one $\mathcal {H}_1$, and those of the Hilbert space of the system two $\mathcal {H}_2$. The basis for the four-dimensional Hilbert space are defined as:

6 $$\begin{aligned}&\vert 0\rangle _1 \otimes \vert 0\rangle _2 \equiv \vert 0, 0\rangle =\vert \varphi _1\rangle \nonumber \\&\vert 0\rangle _1 \otimes \vert 1\rangle _2 \equiv \vert 0, 1\rangle =\vert \varphi _2\rangle \nonumber \\&\vert 1\rangle _1 \otimes \vert 0\rangle _2 \equiv \vert 1, 0\rangle =\vert \varphi _3\rangle \nonumber \\&\vert 1\rangle _1 \otimes \vert 1\rangle _2 \equiv \vert 1, 1\rangle =\vert \varphi _4\rangle. \end{aligned}$$

The state $\vert \psi (0) \rangle$ is a linear combination of the two basis $\vert \varphi _1 \rangle$ and $\vert \varphi _4 \rangle$. In general, it is a statistical ensemble of pure states $\lbrace {p_k,\varphi _k}\rbrace _{k=1,2}$ where each $\vert \varphi _k\rangle$ that occurs with probability $p_k$ can be represented by the orthogonal projector $\rho _k=\vert \varphi _k\rangle \langle \varphi _k\vert$. Hence, the density matrix representation of the initial state can be written as:

7 $$\begin{aligned} \rho =\sum _{k=1,2}p_k\vert \varphi _k\rangle \langle \varphi _k\vert , \end{aligned}$$

where $$\sum _{k=1,2} p_k=1$$.

## Time evolution study of the entangled bipartite system

With *H* defined in Eq. (1) time translation of the initial vector can be represented as $\vert \Psi (t) \rangle \rangle =\hat{U}_I(t)\vert \Psi (0) \rangle \rangle$, where $\hat{U}_I(t)$ is the unitary time evolution operator in interaction picture:

8 $$\begin{aligned} U_I(t)=e^{i(H_S+H_\mathcal {E})t/h}e^{-i(H_S+H_\mathcal {E}+H_{S\mathcal {E}})t/h}, \end{aligned}$$

We suppose that the mRNA and DNA systems placed in separate environments does not interact with each other and hence the action of time evolution operator can be written by:

9 $$\begin{aligned} \vert \Psi (t) \rangle \rangle =\hat{U}_{I,1}(t)\otimes \hat{U}_{I,2}(t)\vert \Psi (0) \rangle \rangle, \end{aligned}$$

${U}_{I,1}(t)=e^{-iH_{S_1\mathcal {E}_1}t/h}$ and ${U}_{I,2}(t)=e^{-iH_{S_2\mathcal {E}_2}t/h}$ represent the time evolution operators for DNA and mRNA strands, respectively. We have used $i=1$ or 2 to show the basis states of the the Hilbert space of DNA as $S_1$ and mRNA as $S_2$, belonging to $\lbrace \vert n\rangle \vert n=0, 1\rbrace$. We obtain the state of the total system at time *t*, expanded in terms of the basis states of the system as follows:

10 $$\begin{aligned} \vert \Psi (t)\rangle \rangle =&\sum _n\vert n\rangle _i{}_{i}\langle n\vert \hat{U}_I(t)\vert \Psi (0)\rangle \rangle \nonumber \\ =&\sum _n\vert n\rangle _1{}_{1}\langle n\vert \hat{U}_{I,1}(t)\vert \Psi (0)\rangle \rangle \vert n\rangle _2{}_{2}\langle n\vert \hat{U}_{I,2}(t)\vert \Psi (0)\rangle \rangle \nonumber \\ =&\sum _n\vert n\rangle \vert \widetilde{\chi _{n_1,n_2}(t)}\rangle. \end{aligned}$$

$\vert \widetilde{\chi _{n_1,n_2}(t)}\rangle$ is the time-dependent coefficients belonging to the Hilbert space of the environment ${\mathcal H}_{\varepsilon _{1}}\otimes {\mathcal H}_{\varepsilon _{2}}$, with the following definition:

11 $$\begin{aligned} \vert \widetilde{\chi _{n_1,n_2}(t)}\rangle ={}_{1}\langle n\vert {}_{2}\langle n\vert \hat{U}_{I,1}(t)\hat{U}_{I,2}(t)\vert \Psi (t)\rangle \rangle, \end{aligned}$$

$n_1$ and $n_2$ in the state $\vert \widetilde{\chi _{n_1,n_2}(t)}\rangle$ specify the states of the DNA as $S_1$ and mRNA as $S_2$, respectively. In order to calculate these coefficients, we resort to the perturbation theory, which can be applied when the system-environment interactions are considered to be weak. Here, regarding $\vert \psi (0) \rangle =\alpha \vert 0\rangle _1\vert 0\rangle _2 +\beta \vert 1\rangle _1\vert 1\rangle _2$ and using Eqs. (10) and (11) we can calculate the coefficients $\vert \widetilde{\chi _{n_1,n_2}(t)}\rangle$ for different values of $n_1$ and $n_2$ as:

12a $$\begin{aligned} \vert \widetilde{\chi _{0,0}(t)}\rangle =&\alpha [{}_{1}\langle 0\vert e^{-iH_{\mathcal {E}_1}t/h}\hat{U}_{I,1}(t)\vert 0\rangle \vert \text {vac}\rangle _1 {}_{2}\langle 0\vert e^{-iH_{\mathcal {E}_2}t/h}\hat{U}_{I,2}(t)\vert 0\rangle _2\vert \text {vac}\rangle _2] \nonumber \\ +&\beta [{}_{1}\langle 0\vert e^{-iH_{\mathcal {E}_1}t/h}\hat{U}_{I,1}(t)\vert 1\rangle \vert \text {vac}\rangle _1 {}_{2}\langle 0\vert e^{-iH_{\mathcal {E}_2}t/h}\hat{U}_{I,2}(t)\vert 1\rangle _2\vert \text {vac}\rangle _2] \end{aligned}$$

12b $$\begin{aligned} \vert \widetilde{\chi _{0,1}(t)}\rangle =&\alpha [{}_{1}\langle 0\vert e^{-iH_{\mathcal {E}_1}t/h}\hat{U}_{I,1}(t)\vert 0\rangle \vert \text {vac}\rangle _1 {}_{2}\langle 1\vert e^{-iH_{\mathcal {E}_2}t/h}\hat{U}_{I,2}(t)\vert 0\rangle _2\vert \text {vac}\rangle _2] \nonumber \\+&\beta [{}_{1}\langle 0\vert e^{-iH_{\mathcal {E}_1}t/h}\hat{U}_{I,1}(t)\vert 1\rangle \vert \text {vac}\rangle _1 {}_{2}\langle 1\vert e^{-iH_{\mathcal {E}_2}t/h}\hat{U}_{I,2}(t)\vert 1\rangle _2\vert \text {vac}\rangle _2] \end{aligned}$$

12c $$\begin{aligned} \vert \widetilde{\chi _{1,0}(t)}\rangle =&\alpha [{}_{1}\langle 1\vert e^{-iH_{\mathcal {E}_1}t/h}\hat{U}_{I,1}(t)\vert 0\rangle \vert \text {vac}\rangle _1 {}_{2}\langle 0\vert e^{-iH_{\mathcal {E}_2}t/h}\hat{U}_{I,2}(t)\vert 0\rangle _2\vert \text {vac}\rangle _2] \nonumber \\ +&\beta [{}_{1}\langle 1\vert e^{-iH_{\mathcal {E}_1}t/h}\hat{U}_{I,1}(t)\vert 1\rangle \vert \text {vac}\rangle _1 {}_{2}\langle 0\vert e^{-iH_{\mathcal {E}_2}t/h}\hat{U}_{I,2}(t)\vert 1\rangle _2\vert \text {vac}\rangle _2] \end{aligned}$$

12d $$\begin{aligned} \vert \widetilde{\chi _{1,1}(t)}\rangle =&\alpha [{}_{1}\langle 1\vert e^{-iH_{\mathcal {E}_1}t/h}\hat{U}_{I,1}(t)\vert 0\rangle \vert \text {vac}\rangle _1 {}_{2}\langle 1\vert e^{-iH_{\mathcal {E}_2}t/h}\hat{U}_{I,2}(t)\vert 0\rangle _2\vert \text {vac}\rangle _2] \nonumber \\ +&\beta [{}_{1}\langle 1\vert e^{-iH_{\mathcal {E}_1}t/h}\hat{U}_{I,1}(t)\vert 1\rangle \vert \text {vac}\rangle _1 {}_{2}\langle 1\vert e^{-iH_{\mathcal {E}_2}t/h}\hat{U}_{I,2}(t)\vert 1\rangle _2\vert \text {vac}\rangle _2]. \end{aligned}$$

We can expand the time-evolution operator ${U}_{I,i}(t)$, regarding the interaction Hamiltonian $H_{S_i\mathcal {E}_i}$ up to the second order to find

13 $$\begin{aligned} \hat{U}_{I,i}(t)&\simeq 1-\dfrac{i}{\tilde{h}} \int _{0}^{t}\text {d}t_1H_{S_i\mathcal {E}_i}(t_1) \nonumber \\&-\dfrac{1}{\tilde{h}^2}\int _{0}^{t}\text {d}t_2\int _{0}^{t_2} \text {d}t_1H_{S_i\mathcal {E}_i}(t_2)H_{S_i\mathcal {E}_i}(t_1), \end{aligned}$$

where the second and third terms of the right-hand side in Eq. (13) are the first and second order correlations, respectively. According to Eqs. (13) and (12) we evaluate the expressions $H_{S_i\mathcal {E}_i}(t_1)\vert \text {vac}\rangle$ and $H_{S_i\mathcal {E}_i}(t_2) H_{S_i\mathcal {E}_i}(t_1)\vert \text {vac}\rangle$ to specify the coefficients $\vert \widetilde{\chi _{n_1,n_2}(t)}\rangle$. Thereby, we arrive at:

14 $$\begin{aligned} \hat{U}_{I,i}(t)\vert \text {vac}\rangle _i \simeq \hat{u}_{\text {vac},i}(t)\vert \text {vac}\rangle _i + \sum _\alpha e^{-i\omega _{\alpha ,i}t}\hat{u}_{\alpha ,i}(t)\vert \alpha \rangle _i .\end{aligned}$$

The detailed forms of the operators $\hat{u}_{\text {vac},i}$ and $\hat{u}_{\alpha ,i}$ are given in Supporting Information (SI). Also, the results for substituting Eq. (14) into (12) to specify the coefficients $\vert \widetilde{\chi _{n_1,n_2}(t)}\rangle$ are given in SI. Here, due to the long equations we avoid to bring all coefficients, and we only bring the coefficient $\vert \widetilde{\chi _{0,0}(t)}\rangle$ here:

15 $$\begin{aligned} \vert \widetilde{\chi _{0,0}(t)}\rangle =\alpha&({}_{1}\langle 0\vert \hat{u}_{\text {vac},1}(t)\vert 0\rangle _1\vert \text {vac}\rangle _1+\sum _\alpha e^{-i\omega _{\alpha ,1}t}{}_{1}\langle 0\vert \hat{u}_{\alpha ,1}(t)\vert 0\rangle _1\vert \alpha \rangle _1)\nonumber \\&({}_{2}\langle 0\vert \hat{u}_{\text {vac},2}(t)\vert 0\rangle _2\vert \text {vac}\rangle _2+\sum _\alpha e^{-i\omega _{\alpha ,2}t}{}_{2}\langle 0\vert \hat{u}_{\alpha ,2}(t)\vert 0\rangle _2\vert \alpha \rangle _2)\nonumber \\ +\beta&({}_{1}\langle 0\vert \hat{u}_{\text {vac},1}(t)\vert 1\rangle _1\vert \text {vac}\rangle _1+\sum _\alpha e^{-i\omega _{\alpha ,1}t}{}_{1}\langle 0\vert \hat{u}_{\alpha ,1}(t)\vert 1\rangle _1\vert \alpha \rangle _1)\nonumber \\&({}_{2}\langle 0\vert \hat{u}_{\text {vac},2}(t)\vert 1\rangle _2\vert \text {vac}\rangle _2+\sum _\alpha e^{-i\omega _{\alpha ,2}t}{}_{2}\langle 0\vert \hat{u}_{\alpha ,2}(t)\vert 1\rangle _2\vert \alpha \rangle _2) ,\end{aligned}$$

At last, the problem of calculation of the coefficients $\vert \widetilde{\chi _{n_1,n_2}(t)}\rangle$ is reduced to finding the matrix elements of the operators $\hat{u}_{\text {vac},i}$, $\hat{u}_{\alpha ,i}$. In this sense, some parity considerations will be useful to estimate the matrix elements of the operators:

16a $$\begin{aligned} {}_{i}\langle m\vert \hat{u}_{\text {vac},i}\vert n\rangle _i = {\left\{ \begin{array}{ll} \textit{zero} &{}: m-n \text { is odd} \\ \textit{non-zero} &{}: m-n \text { is even} \end{array}\right. } \end{aligned}$$

16b $$\begin{aligned} {}_{i}\langle m\vert \hat{u}_{\alpha ,i}\vert n\rangle _i= {\left\{ \begin{array}{ll} \textit{zero} &{}: m-n \text { is even} \\ \textit{non-zero} &{}: m-n \text { is odd} \end{array}\right. } \end{aligned}$$

Using the parity rules in Eq. (16) we can simplify $\vert \widetilde{\chi _{0,0}(t)}\rangle$ to obtain the following expressions:

17a $$\begin{aligned} \vert \widetilde{\chi _{0,0}(t)}\rangle =\alpha&({}_{1}\langle 0\vert \hat{u}_{\text {vac},1}(t)\vert 0\rangle _1\vert \text {vac}\rangle _1{}_{2}\langle 0\vert \hat{u}_{\text {vac},2}(t)\vert 0\rangle _2\vert \text {vac}\rangle _2)\nonumber \\ +\beta&(\sum _\alpha e^{-i\omega _{\alpha ,1}t}e^{-i\omega _{\alpha ,2}t}{}_{1}\langle 0\vert \hat{u}_{\alpha ,1}(t)\vert 1\rangle _1\vert \alpha \rangle _1{}_{2}\langle 0\vert \hat{u}_{\alpha ,2}(t)\vert 1\rangle _2\vert \alpha \rangle _2) \end{aligned}$$

17b $$\begin{aligned} \vert \widetilde{\chi _{0,1}(t)}\rangle =\alpha&({}_{1}\langle 0\vert \hat{u}_{\text {vac},1}(t)\vert 0\rangle _1\vert \text {vac}\rangle _1\sum _\alpha e^{-i\omega _{\alpha ,2}t}{}_{2}\langle 1\vert \hat{u}_{\alpha ,2}(t)\vert 0\rangle _2\vert \alpha \rangle _2)\nonumber \\ +\beta&(\sum _\alpha e^{-i\omega _{\alpha ,1}t}{}_{1}\langle 0\vert \hat{u}_{\alpha ,1}(t)\vert 1\rangle _1\vert \alpha \rangle _1{}_{2}\langle 0\vert \hat{u}_{\text {vac},2}(t)\vert 0\rangle _2\vert \text {vac}\rangle _2) \end{aligned}$$

17c $$\begin{aligned} \vert \widetilde{\chi _{1,0}(t)}\rangle =\alpha&(\sum _\alpha e^{-i\omega _{\alpha ,1}t}{}_{1}\langle 1\vert \hat{u}_{\alpha ,1}(t)\vert 0\rangle _1\vert \alpha \rangle _1{}_{2}\langle 0\vert \hat{u}_{\text {vac},2}(t)\vert 0\rangle _2\vert \text {vac}\rangle _2)\nonumber \\ +\beta&({}_{1}\langle 1\vert \hat{u}_{\text {vac},1}(t)\vert 1\rangle _1\vert \text {vac}\rangle _1\sum _\alpha e^{-i\omega _{\alpha ,2}t}{}_{2}\langle 0\vert \hat{u}_{\alpha ,2}(t)\vert 1\rangle _2\vert \alpha \rangle _2) \end{aligned}$$

17d $$\begin{aligned} \vert \widetilde{\chi _{1,1}(t)}\rangle =\alpha&(\sum _\alpha e^{-i\omega _{\alpha ,1}t}e^{-i\omega _{\alpha ,2}t}{}_{1}\langle 1\vert \hat{u}_{\alpha ,1}(t)\vert 0\rangle _1\vert \alpha \rangle _1{}_{2}\langle 1\vert \hat{u}_{\alpha ,2}(t)\vert 0\rangle _2\vert \alpha \rangle _2) \nonumber \\ +\beta&({}_{1}\langle 1\vert \hat{u}_{\text {vac},1}(t)\vert 1\rangle _1\vert \text {vac}\rangle _1{}_{2}\langle 1\vert \hat{u}_{\text {vac},2}(t)\vert 1\rangle _2\vert \text {vac}\rangle _2) \end{aligned}$$

Using the coefficients specified in Eqs. (17a) to (17d) the total state of the system at time *t* would be

18 $$\begin{aligned} \vert \Psi (t)\rangle \rangle =&\sum _n e^{-iE_{n,1}t/h}e^{-iE_{n,2}t/h}\vert n\rangle _1\vert n\rangle _2\vert \widetilde{\chi _{n_1,n_2}(t)}\rangle \nonumber \\ =&\sum _n e^{-iE_{0,1}t/h}e^{-iE_{0,2}t/h}\vert 0\rangle _1\vert 0\rangle _2\vert \widetilde{\chi _{0,0}(t)}\rangle \nonumber \\ +&\sum _n e^{-iE_{0,1}t/h}e^{-iE_{1,2}t/h}\vert 0\rangle _1\vert 1\rangle _2\vert \widetilde{\chi _{0,1}(t)}\rangle \nonumber \\ +&\sum _n e^{-iE_{1,1}t/h}e^{-iE_{0,2}t/h}\vert 1\rangle _1\vert 0\rangle _2\vert \widetilde{\chi _{1,0}(t)}\rangle \nonumber \\ +&\sum _n e^{-iE_{1,1}t/h}e^{-iE_{1,2}t/h}\vert 1\rangle _1\vert 1\rangle _2\vert \widetilde{\chi _{1,1}(t)}\rangle .\end{aligned}$$

Evaluating the non-zero matrix elements in Eq. (17) and then substituting the results into (18), enables us to obtain the probability of finding the system in initial state as:

19 $$\begin{aligned} \vert \langle \Psi (0)\vert \Psi (t)\rangle \vert ^2=\alpha ^4+\beta ^4 e^{-\Gamma _1 t}e^{-\Gamma _2 t}+2\alpha ^2\beta ^2e^{-\Gamma _1 t/2}e^{-\Gamma _2 t/2}\cos (\Delta _1+\Delta _2)t .\end{aligned}$$

**Figure 2:**
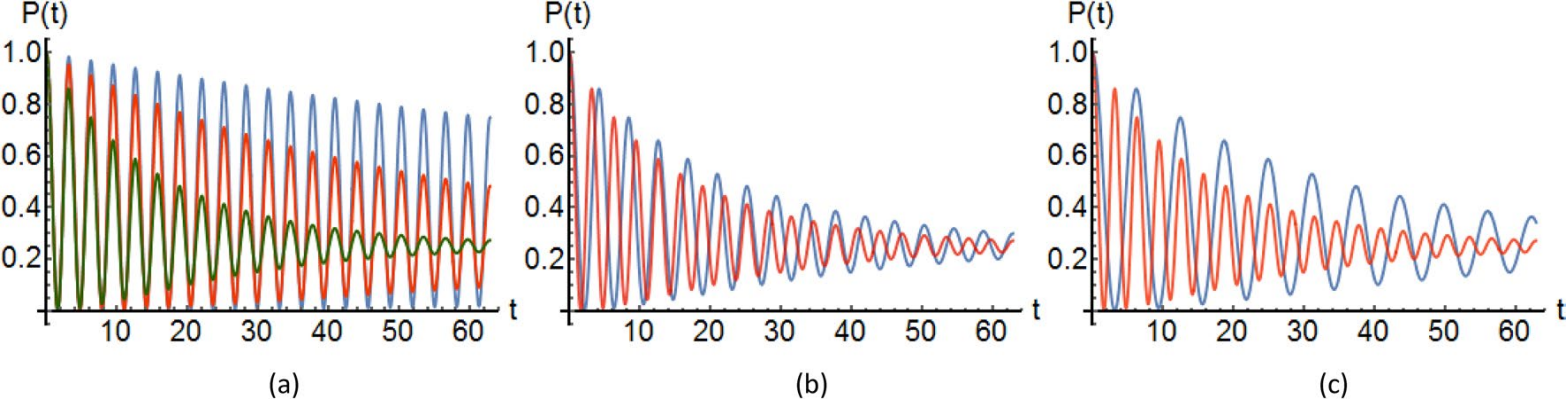
The dynamics of the probability for initial entangled state of bipartite system of DNA and mRNA, (**a**) plot of $P(\Psi =\Psi (0))$ as a function of *t* (dimensionless) with the values $\Delta _1=\Delta _2=\Delta =1$, $\Gamma _1=\Gamma _2=\Gamma$ and $\Gamma _{\textit{blue}}<\Gamma _{\textit{red}}<\Gamma _{\textit{green}}$ (**b**) same plot as (**a**) with the values $\Delta _1=\Delta _2$, $\Gamma _1=\Gamma _2$ for *red* curve and $\Delta _1>\Delta _2$, $\Gamma _1>\Gamma _2$ for *blue* curve, (**c**) same plot as (**b**) but with the values $\Delta _1\gg \Delta _2$ and $\Gamma _1\gg \Gamma _2$ for *blue* curve. See exact values in SI.

Figure 2 showes the dynamics of the probability for initial entangled state of bipartite system composed from DNA and mRNA. In this figure, the probability is plotted for different tunneling amplitudes and system-environment interaction strengths.

## The concurrence of the bipartite system

Here, we use the obtaind result of the previous section to evaluate the degree of entanglement of the system as a function of time. One striking measure to evaluate the degree of entanglement is concurrence that takes the value 1 for maximal entangled and 0 for unentangled systems. The concurrence of two qubits introduced by Hill and Wootters represents an appropriate option to answer the question of what extent the given quantum state is entangled^40,41^? According to^40,41^, for a given two-qubit density operator $\rho$, the measure of concurrence $C(\rho )$ is calculated as:

20 $$\begin{aligned} C(\rho )=\text {max}\lbrace 0, \sqrt{\lambda _1}-\sqrt{\lambda _2}-\sqrt{\lambda _3}-\sqrt{\lambda _4}\rbrace, \end{aligned}$$

where the $\lambda _i$ parameters, sorted in descending order, are the eigenvalues of the matrix $\rho (\sigma _y\otimes \sigma _y)\rho ^*(\sigma _y\otimes \sigma _y)$ and $\rho ^*$ is the complex conjugation of the density matrix $\rho$. For the given $\vert \Psi (t)\rangle$, the density operator $\rho$ of the whole system is the defined as $\rho =\vert \Psi (t)\rangle \langle \Psi (t)\vert$. Here, we calculate $\rho _S:=Tr_{\mathcal {E}_1,\mathcal {E}_2}\rho$ to gain information about entangled system composed of DNA and mRNA qubits. We obtain $\rho _S$ as

21 $$\begin{aligned} \rho _S=Tr_{\mathcal {E}_1,\mathcal {E}_2}\rho =&\vert 0\rangle _1\vert 0\rangle _2\vert \widetilde{\chi _{0,0}(t)}\rangle \langle \widetilde{\chi _{0,0}(t)}\vert {}_{2}\langle 0\vert {}_{1}\langle 0\vert \nonumber \\+&e^{+i\Delta _{1}t}e^{+i\Delta _{2}t}\vert 0\rangle _1\vert 0\rangle _2\vert \widetilde{\chi _{0,0}(t)}\rangle \langle \widetilde{\chi _{1,1}(t)}\vert {}_{2}\langle 1\vert {}_{1}\langle 1\vert \nonumber \\ +&\vert 0\rangle _1\vert 1\rangle _2\vert \widetilde{\chi _{0,1}(t)}\rangle \langle \widetilde{\chi _{0,1}(t)}\vert {}_{2}\langle 1\vert {}_{1}\langle 0\vert \nonumber \\ +&e^{+i\Delta _{1}t}e^{-i\Delta _{2}t}\vert 0\rangle _1\vert 1\rangle _2\vert \widetilde{\chi _{0,1}(t)}\rangle \langle \widetilde{\chi _{1,0}(t)}\vert {}_{2}\langle 0\vert {}_{1}\langle 1\vert \nonumber \\ +&e^{-i\Delta _{1}t}e^{+i\Delta _{2}t}\vert 1\rangle _1\vert 0\rangle _2\vert \widetilde{\chi _{1,0}(t)}\rangle \langle \widetilde{\chi _{0,1}(t)}\vert {}_{2}\langle 1\vert {}_{1}\langle 0\vert \nonumber \\ +&\vert 1\rangle _1\vert 0\rangle _2\vert \widetilde{\chi _{1,0}(t)}\rangle \langle \widetilde{\chi _{1,0}(t)}\vert {}_{2}\langle 0\vert {}_{1}\langle 1\vert \nonumber \\ +&e^{-i\Delta _{1}t}e^{+i\Delta _{2}t}\vert 1\rangle _1\vert 1\rangle _2\vert \widetilde{\chi _{1,1}(t)}\rangle \langle \widetilde{\chi _{0,0}(t)}\vert {}_{2}\langle 0\vert {}_{1}\langle 0\vert \nonumber \\ +&\vert 1\rangle _1\vert 1\rangle _2\vert \widetilde{\chi _{1,1}(t)}\rangle \langle \widetilde{\chi _{1,1}(t)}\vert {}_{2}\langle 1\vert {}_{1}\langle 1\vert \end{aligned}$$

By evaluating the expressions $\vert \widetilde{\chi _{n_1,n_2}(t)}\rangle \langle \widetilde{\chi _{n_1,n_2}(t)}\vert$ in Eq. (21) we obtain the density matrix $\rho _S$ in terms of the matrix elements of the components of the time evolution operator, $\hat{u}_\text {vac}(t)$ and $\hat{u}_\alpha (t)$ as follows

22 $$\begin{aligned} \rho _S= \begin{bmatrix} \Lambda _{11} &{} 0 &{} 0 &{} \Lambda _{14} \\ 0 &{} \Lambda _{22} &{} \Lambda _{23} &{} 0 \\ 0 &{} \Lambda _{32} &{} \Lambda _{33} &{} 0 \\ \Lambda _{41} &{} 0 &{} 0 &{} \Lambda _{44} \end{bmatrix} \end{aligned}$$

where the matrix elements $\Lambda _{ij}$ are calculated in SI.

Accordingly, we obtain all non-vanishing matrix elements of the operators $\hat{u}_\text {vac}(t)$ and $\hat{u}_\alpha (t)$ as

23a $$\begin{aligned} \langle 0 \vert \hat{u}_{\text {vac},i}(t)\vert 0 \rangle&\simeq \text {exp} [ -\dfrac{\text {i}}{\tilde{h}}\lbrace t\delta E_{0,i} - \vert f_{10,i}\vert ^2 F_{+,i}(t)\rbrace ] \end{aligned}$$

23b $$\begin{aligned} \langle 1 \vert \hat{u}_{\text {vac},i}(t)\vert 1 \rangle&\simeq \text {exp} [ -\dfrac{\text {i}}{\tilde{h}}\lbrace t\delta E_{1,i} - \vert f_{01,i}\vert ^2 F_{-,i}(t)\rbrace ] \end{aligned}$$

23c $$\begin{aligned} \langle 0 \vert \hat{u}_{\alpha ,i}(t)\vert 1 \rangle&=\langle 1 \vert \hat{u}_{\alpha ,i}(t)\vert 0 \rangle ^* =\dfrac{2\pi \text {i}}{\sqrt{2\tilde{h}}}\bar{\gamma }_{\alpha ,i} f_{01,i}\left( \dfrac{ 1}{\pi }\right) \dfrac{\sin {(\omega _i +\Delta _i) t/2}}{\omega _i +\Delta _i}e^{\text {i}(\omega _i + \Delta _i)t/2} \end{aligned}$$

where $F_{\pm ,i}(t)=-\pi ^{(-1)} \mathcal {P}\int _0^\infty d\omega _i J(\omega _i)\dfrac{\sin {(\omega _i \pm \Delta _i) t}}{(\omega _i \pm \Delta _i)^2}$. Here the symbol $\mathcal {P}$ denotes that the integral is a principal-value integral, $\Delta _i:=\dfrac{E_{1,i}-E_{0,i}}{\tilde{h}}$ is called the tunnel splitting of the ground-state energy. It is worth to note that the function $J(\omega )$ namely the spectral function in the literature^38^, has the form $J(\omega ):=\dfrac{\pi }{2}\lbrace \bar{\gamma }(\omega )\rbrace ^2D(\omega )$. The function $D(\omega )$ presents the frequency distribution of the environmental oscillators and $J(\omega )$ expresses the corresponding distribution weighted by the function $\lbrace \bar{\gamma }(\omega )\rbrace ^2$ which describes the interaction strength. In our regime, $D(\omega )$ is defined as $D(\omega ):=\dfrac{1}{2\pi t}\lbrace \dfrac{\sin (\omega t/2)}{\omega /2}\rbrace ^2$. Substituting non-vanishing matrix elements of the operators $\hat{u}_\text {vac}(t)$ and $\hat{u}_\alpha (t)$ into the expressions obtaind for $\Lambda _{ij}$ (see SI) and simplifying consequent relations we have

24 $$\begin{aligned} \Lambda _{11}=&\alpha ^2 \nonumber \\ \Lambda _{14}=&\alpha \beta ^* e^{-\Gamma _{1}t/2}e^{-\Gamma _{2}t/2}e^{+i\Delta _{1}t}e^{+i\Delta _{2}t}\nonumber \\ \Lambda _{22}=&\beta ^2 \Gamma _1 t e^{-\Gamma _{2}t/2}\simeq \beta ^2 (1-e^{\Gamma _1 t}) e^{-\Gamma _{2}t}\nonumber \\ \Lambda _{23}\simeq&0\nonumber \\ \Lambda _{32}\simeq&0\nonumber \\ \Lambda _{22}=&\beta ^2 \Gamma _2 t e^{-\Gamma _{1}t/2}\simeq \beta ^2 e^{-\Gamma _{1}t/2}(1-e^{\Gamma _1 t})\nonumber \\ \Lambda _{41}=&\beta \alpha ^* e^{-\Gamma _{1}t/2}e^{-\Gamma _{2}t/2}e^{-i\Delta _{1}t}e^{-i\Delta _{2}t}\nonumber \\ \Lambda _{44}=&\beta ^2 e^{-\Gamma _{1}t}e^{-\Gamma _{2}t} \end{aligned}$$

The eigenvalues of the matrix $\rho (\sigma _y\otimes \sigma _y)\rho ^*(\sigma _y\otimes \sigma _y)$ read as follows

25 $$\begin{aligned} \lambda _1&=\lambda _2=\Lambda _{22}\Lambda _{33}\nonumber \\ \lambda _3&=\dfrac{1}{2}{[(2\Lambda _{11}\Lambda _{44})^{1/2}-(2\Lambda _{14}\Lambda _{41})^{1/2}]}\nonumber \\ \lambda _4&=\dfrac{1}{2}{[(2\Lambda _{11}\Lambda _{44})^{1/2}+(2\Lambda _{14}\Lambda _{41})^{1/2}]} \end{aligned}$$

Therefore, according to Eq. (20) we find the concurrence of the bipartite entangled system as

26 $$\begin{aligned} C(\rho )=2\alpha \beta e^{-\Gamma _{1}t/2}e^{-\Gamma _{2}t/2}-2\beta ^2 e^{-\Gamma _{1}t/2}e^{-\Gamma _{2}t/2}(1-e^{\Gamma _1 t})^{1/2}(1-e^{\Gamma _2 t})^{1/2}. \end{aligned}$$

**Figure 3:**
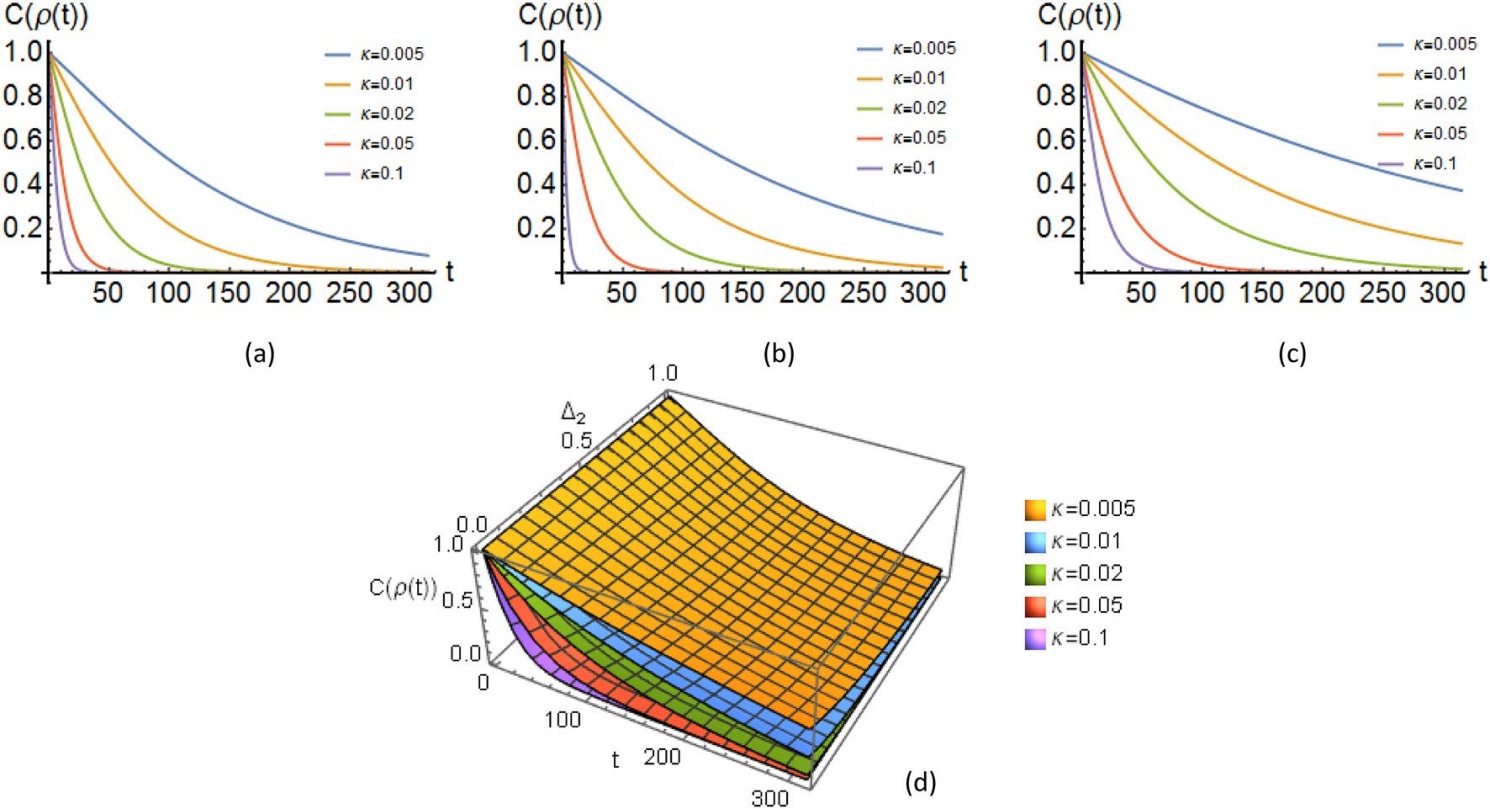
The time-evolution of the concurrence associated to the mRNA-DNA entangled state, (**a**) plot of $C(\rho )$ as a function of *t* (dimensionless) with the values $\alpha =\cos {\pi /4}$, $\beta =\sin {\pi /4}$, $\Delta _1=\Delta _2=1$, $\Gamma _1=\Gamma _2$ and also $\Gamma _1$ ($\Gamma _2$) is equal to $\kappa \Delta _1$ ($\kappa \Delta _2$), (**b**) same as (**a**) but $\Delta _1>\Delta _2$, (**c**) same as (**a**) but $\Delta _1\gg \Delta _2$, (**d**) 3D plot of $C(\rho )$ as a function of *t* and $\Delta _2$ with same values as (**a**) but $\Delta _2$ changes from 0 to 1. See SI for exact values.

**Figure 4:**
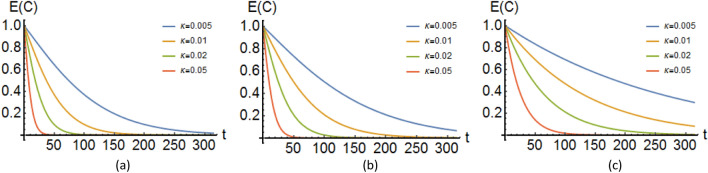
The time-evolution of the entanglement of formation associated to the bipartite mRNA-DNA entangled state, (**a**) plot of *E*(*C*) as a function of *t* (dimensionless) with the values $\alpha =\cos {\pi /4}$, $\beta =\sin {\pi /4}$, $\Delta _1=\Delta _2=1$, $\Gamma _1=\Gamma _2$ and also $\Gamma _1$ ($\Gamma _2$) is equal to $\kappa \Delta _1$ ($\kappa \Delta _2$), (**b**) same as (**a**) but $\Delta _1>\Delta _2$, (**c**) same as (**a**) but $\Delta _1\gg \Delta _2$. See SI for exact values.

Figure 3 shows the behavior of the concurrence as a function of time for the equally weighted entangled state (5), i.e. $\Psi _{\alpha =\beta =1/\sqrt{2}}$ considered as initial conditions for the bipartite state. The state $\Psi (t=0)$ corresponds to the situation in which the mRNA-DNA system has the probability $\cos {(\dfrac{\pi }{4})}$ to be in the state $\vert \varphi _1\rangle =\vert 0,0\rangle$ and $\sin {(\dfrac{\pi }{4})}$ to be in the state $\vert \varphi _4\rangle =\vert 1,1\rangle$. That is, the repeat of measurement will give equally the states $\vert \varphi _1\rangle =\vert 0,0\rangle$ and $\vert \varphi _4\rangle =\vert 1,1\rangle$ at times the system is checked whether it is in the examined state or not. Remark that in this case the correlation obtained from the interaction process is present and the state $\Psi (t=0)$ encodes a strong entanglement between the components of the entire system. In this figure, $C(\rho )$ is plotted for different tunneling amplitudes and system-environment interaction strengths. In Figure 3, we can infer that the concurrence curve for the state $\Psi (t=0)$ begins from a maximally entangled condition, where concurrence is equal to one, and after a while goes to the less entangled condition. For large values of $\Delta _i$s and $\Gamma _i$s, the concurrence approaches zero faster than the region in which their amounts are negligible. For a situation in which the entangled state confront with environments with different values of $\Delta _i$ and $\Gamma _i$, the decoherence process occurs slowly. Thus the entanglement between mRNA and DNA survives for a longer time. In contrast, the environments with similar properties (the near values for $\Delta _i$s and $\Gamma _i$s) cause more rapid decoherence. Note that here decoherence can stabilize the wave function in the mutated state and thus generate colonies from the mutated states as a representation for tumor formation.

Moreover, employing an explicit expression for $\Gamma _i$ as $\Gamma (\tau )= \dfrac{1}{\pi \tilde{h}}\vert f_{01} \vert ^2\eta {{\int _0}^\infty } d\omega \left( \dfrac{\omega }{\omega _c}\right) ^{s-1}\omega \exp (-\omega /\omega _c)\dfrac{1}{ t}\lbrace \dfrac{\sin (\omega t/2)}{\omega /2}\rbrace ^2$, can connect this process to Ohmic behavior of the environments. In this relation, *s* characterizes the Ohmicity of the environment, $\omega _c$ is the cutoff frequency, and $\eta$ is a positive constant to quantify the system-environment interaction strength.

If $\Psi _{\alpha \ne \beta \ne 1/\sqrt{2}}$ is the initial condition, then we see a very similar process, but the concurrence starts with a smaller value, admits that entanglement is going to be shaped and has not been completed, yet).

Let us now attend to another measure of entanglement. For a given mixed state $\rho$ of two quantum systems $S_1$ and $S_2$, we consider all possible decomposition of pure states of $\rho$ in terms of an ensemble of pure states $\vert \psi _i\rangle$ and associated probabilities $p_i$, that is $\rho = \sum _i p_i\vert \psi _i\rangle \langle \psi _i\vert$, then the entanglement of formation $E(\rho )$ can be defined as the average entanglement of the pure states of the decomposition, minimized over all decomposition of $\rho$:

27 $$\begin{aligned} E(\rho )=\text {min}\sum _i p_i E(\psi _i). \end{aligned}$$

It has been verified that this entanglement of formation can be written as

28 $$\begin{aligned} E(\psi )={E} (C(\psi _i)). \end{aligned}$$

where the function $C(\psi _i)$ is concurrence of $\psi _i$ and and the function $\textit{E}$ is given by

29 $$\begin{aligned} E(C)&=h\left( \dfrac{1+\sqrt{1-C^{2}}}{2} \right) ; \nonumber \\ h(x)&= -x \log _2 x - (1-x) \log _2 (1-x). \end{aligned}$$

Using the Eq. (28) we can obtain the entanglement of formation *E*(*C*) for the calculated expression of the cuncurrence. Figure 4 demonstrates the variation of the measure *E*(*C*) with time for different values of $\Delta _i$ and $\Gamma _i$. According to Fig. 4, as we expect the entanglement of formation *E*(*C*) shows a similar behavior to the concurrence which is demonstrated in Fig. 3.

## Conclusion

In the present work, we studied the environment-induced decoherence for mRNA-DNA-error correllation created due to the base tautomery, considering the mRNA-DNA system as entangled bipartite system, where each part coupled to a different bosonic environments. The time-evolution of the energy states of the pair has been studied entangled biomolecules has been studied using time-dependent perturbation theory. The concurrence of these systems calculated in order to determine how extent the components are entangled. We have found that the mRNA-DNA-error correlation depends on the interaction strength of the each component and the corresponding environment. The parameters $\Gamma _i$ and $\Delta _i$ which represent the system-environment interaction and tunneling strength, respectively, are effective factors in EID process. When the interaction strength for both parts are strong, the generated entanglement cannot survive for long time. In fact, EID will suppress one of the cat-like states, but another states will be stabilized by EID. If the mutated state selectid as stabilized state, in some condition (presence of special substrate) colonies of mutayed cells will be generated. Some other questions like the dependence of the entanglement with the self-measurement process in presence of a substrate, controling time evolution dynamics and EID process are going to be discussed in future works. Moreover, in this work, as previous studies, we have treated helicase opening to come after the proton transfer. But there is not reasonable evidence that the helicase does not induce the decoherence event. There is the possibility that the helicase, in interaction with DNA, acts as a measuring device. Consequently, two macroscopically distinguishable states of the normal and tautomeric forms may be generated after the complete separation of strands. In other words, the helicase may open Schrödinger’s box as an observer. This issue needs to be considered and addressed. Further investigations on the effect of the separation distance between nucleotides on the amount of coherence of the parent DNA nucleotides and the consequent mutational event should be considered and performed in future works.

### Supplementary Information

Supplementary Information.

## Data Availability

All theoretical findings during the current study are not publicly available due to the extended calsulations but are available from the corresponding author on reasonable request.
