# Author Correction: Environment assisted quantum model for studying RNA-DNA-error correlation created due to the base tautomery

**DOI:** 10.1038/s41598-023-48156-8

**Published:** 2023-11-27

**Authors:** Fatemeh Ghasemi, Arash Tirandaz

**Affiliations:** 1 Department of Energy Engineering, Sharif University of Technology, P.O. Box 11365-9516, Tehran, Iran; 2 Department of Chemistry, Bu-Ali Sina University, Hamedan, Iran

Correction to: *Scientific Reports* 10.1038/s41598-023-38019-7, published online 04 July 2023

In the original version of this Article, author Arash Tirandaz was omitted as a corresponding author. Correspondence and requests for materials should also be addressed to a.tirandaz@basu.ac.ir.

The original Article has been corrected.

