# Supplementary material for: Environment assisted quantum model for studying RNA-DNA-error correlation created due to the base tautomery
Source: Sci Rep. 2023 Jul 4;13:10788. doi: 10.1038/s41598-023-38019-7 (PMC10319750; doi:10.1038/s41598-023-38019-7)
Supplement: Supplementary file 1 — Supplementary Information. [file 41598_2023_38019_MOESM1_ESM.pdf]

# Environment Assisted Quantum Model for Studying RNA-DNA-error Correlation Created Due to the Base Tautomery

Fatemeh Ghasemi and Arash Tirandaz

# I. APPENDIX

The operators $\hat{u}_{\text{vac}}(t)$ and $\hat{u}_\alpha(t)$ are defined as

$$\begin{aligned}\hat{u}_{\text{vac}}(t) &:= 1 - \frac{\mathrm{i}}{\tilde{h}} \int_0^t \mathrm{d}t_1 \delta\hat{V}(t_1) \\ &\quad - \frac{1}{2\tilde{h}} \sum_\alpha \int_0^t \mathrm{d}t_2 \int_0^{t_2} \mathrm{d}t_1 \hat{f}_\alpha(t_2) {\omega_\alpha}^3 \mathrm{e}^{-\mathrm{i}(t_2-t_1)\omega_\alpha} \hat{f}_\alpha(t_1) \end{aligned} \tag{1}$$

$$\hat{u}_\alpha(t) := \frac{\mathrm{i}}{\sqrt{2\tilde{h}}} \int_0^t \mathrm{d}t_1 \hat{f}_\alpha(t_1) {\omega_\alpha}^{3/2} \mathrm{e}^{-\mathrm{i}\omega_\alpha t_1} \tag{2}$$

Accordingly, for $|\ \chi^{\sim}_{n_1,n_2}\rangle$ one has:

$$\begin{aligned}|\widetilde{\chi_{0,0}(t)}\rangle =&\alpha[{}_1\langle 0|e^{-iH_{\mathcal{E}_1}t/h}\hat{U}_{I,1}(t)|0\rangle|\text{vac}\rangle_{1\,2}\langle 0|e^{-iH_{\mathcal{E}_2}t/h}\hat{U}_{I,2}(t)|0\rangle_2|\text{vac}\rangle_2] \\ &+\beta[{}_1\langle 0|e^{-iH_{\mathcal{E}_1}t/h}\hat{U}_{I,1}(t)|1\rangle|\text{vac}\rangle_{1\,2}\langle 0|e^{-iH_{\mathcal{E}_2}t/h}\hat{U}_{I,2}(t)|1\rangle_2|\text{vac}\rangle_2] \end{aligned} \tag{3a}$$

$$\begin{aligned}|\widetilde{\chi_{0,1}(t)}\rangle =&\alpha[{}_1\langle 0|e^{-iH_{\mathcal{E}_1}t/h}\hat{U}_{I,1}(t)|0\rangle|\text{vac}\rangle_{1\,2}\langle 1|e^{-iH_{\mathcal{E}_2}t/h}\hat{U}_{I,2}(t)|0\rangle_2|\text{vac}\rangle_2] \\ &+\beta[{}_1\langle 0|e^{-iH_{\mathcal{E}_1}t/h}\hat{U}_{I,1}(t)|1\rangle|\text{vac}\rangle_{1\,2}\langle 1|e^{-iH_{\mathcal{E}_2}t/h}\hat{U}_{I,2}(t)|1\rangle_2|\text{vac}\rangle_2] \end{aligned} \tag{3b}$$

$$\begin{aligned}|\widetilde{\chi_{1,0}(t)}\rangle =&\alpha[{}_1\langle 1|e^{-iH_{\mathcal{E}_1}t/h}\hat{U}_{I,1}(t)|0\rangle|\text{vac}\rangle_{1\,2}\langle 0|e^{-iH_{\mathcal{E}_2}t/h}\hat{U}_{I,2}(t)|0\rangle_2|\text{vac}\rangle_2] \\ &+\beta[{}_1\langle 1|e^{-iH_{\mathcal{E}_1}t/h}\hat{U}_{I,1}(t)|1\rangle|\text{vac}\rangle_{1\,2}\langle 0|e^{-iH_{\mathcal{E}_2}t/h}\hat{U}_{I,2}(t)|1\rangle_2|\text{vac}\rangle_2] \end{aligned} \tag{3c}$$

$$\begin{aligned}|\widetilde{\chi_{1,1}(t)}\rangle =&\alpha[{}_1\langle 1|e^{-iH_{\mathcal{E}_1}t/h}\hat{U}_{I,1}(t)|0\rangle|\text{vac}\rangle_{1\,2}\langle 1|e^{-iH_{\mathcal{E}_2}t/h}\hat{U}_{I,2}(t)|0\rangle_2|\text{vac}\rangle_2] \\ &+\beta[{}_1\langle 1|e^{-iH_{\mathcal{E}_1}t/h}\hat{U}_{I,1}(t)|1\rangle|\text{vac}\rangle_{1\,2}\langle 1|e^{-iH_{\mathcal{E}_2}t/h}\hat{U}_{I,2}(t)|1\rangle_2|\text{vac}\rangle_2] \end{aligned} \tag{3d}$$

The coefficients $|\widetilde{\chi_{n_1,n_2}(t)}\rangle$ using Eqs. (13) in main text and (3a) in appendix take the following forms

$$
\begin{aligned}
|\widetilde{\chi_{0,0}(t)}\rangle = {} & \alpha({}_1\langle 0|\hat{u}_{\mathrm{vac},1}(t)|0\rangle_1|\mathrm{vac}\rangle_1 + \sum_\alpha e^{-i\omega_{\alpha,1}t}{}_1\langle 0|\hat{u}_{\alpha,1}(t)|0\rangle_1|\alpha\rangle_1) \\
& ({}_2\langle 0|\hat{u}_{\mathrm{vac},2}(t)|0\rangle_2|\mathrm{vac}\rangle_2 + \sum_\alpha e^{-i\omega_{\alpha,2}t}{}_2\langle 0|\hat{u}_{\alpha,2}(t)|0\rangle_2|\alpha\rangle_2) \\
& +\beta({}_1\langle 0|\hat{u}_{\mathrm{vac},1}(t)|1\rangle_1|\mathrm{vac}\rangle_1 + \sum_\alpha e^{-i\omega_{\alpha,1}t}{}_1\langle 0|\hat{u}_{\alpha,1}(t)|1\rangle_1|\alpha\rangle_1) \\
& ({}_2\langle 0|\hat{u}_{\mathrm{vac},2}(t)|1\rangle_2|\mathrm{vac}\rangle_2 + \sum_\alpha e^{-i\omega_{\alpha,2}t}{}_2\langle 0|\hat{u}_{\alpha,2}(t)|1\rangle_2|\alpha\rangle_2) \qquad (4a) \\
|\widetilde{\chi_{0,1}(t)}\rangle = {} & \alpha({}_1\langle 0|\hat{u}_{\mathrm{vac},1}(t)|0\rangle_1|\mathrm{vac}\rangle_1 + \sum_\alpha e^{-i\omega_{\alpha,1}t}{}_1\langle 0|\hat{u}_{\alpha,1}(t)|0\rangle_1|\alpha\rangle_1) \\
& ({}_2\langle 1|\hat{u}_{\mathrm{vac},2}(t)|0\rangle_2|\mathrm{vac}\rangle_2 + \sum_\alpha e^{-i\omega_{\alpha,2}t}{}_2\langle 1|\hat{u}_{\alpha,2}(t)|0\rangle_2|\alpha\rangle_2) \\
& +\beta({}_1\langle 0|\hat{u}_{\mathrm{vac},1}(t)|1\rangle_1|\mathrm{vac}\rangle_1 + \sum_\alpha e^{-i\omega_{\alpha,1}t}{}_1\langle 0|\hat{u}_{\alpha,1}(t)|1\rangle_1|\alpha\rangle_1) \\
& ({}_2\langle 1|\hat{u}_{\mathrm{vac},2}(t)|1\rangle_2|\mathrm{vac}\rangle_2 + \sum_\alpha e^{-i\omega_{\alpha,2}t}{}_2\langle 1|\hat{u}_{\alpha,2}(t)|1\rangle_2|\alpha\rangle_2) \qquad (4b) \\
|\widetilde{\chi_{1,0}(t)}\rangle = {} & \alpha({}_1\langle 1|\hat{u}_{\mathrm{vac},1}(t)|0\rangle_1|\mathrm{vac}\rangle_1 + \sum_\alpha e^{-i\omega_{\alpha,1}t}{}_1\langle 1|\hat{u}_{\alpha,1}(t)|0\rangle_1|\alpha\rangle_1) \\
& ({}_2\langle 0|\hat{u}_{\mathrm{vac},2}(t)|0\rangle_2|\mathrm{vac}\rangle_2 + \sum_\alpha e^{-i\omega_{\alpha,2}t}{}_2\langle 0|\hat{u}_{\alpha,2}(t)|0\rangle_2|\alpha\rangle_2) \\
& +\beta({}_1\langle 1|\hat{u}_{\mathrm{vac},1}(t)|1\rangle_1|\mathrm{vac}\rangle_1 + \sum_\alpha e^{-i\omega_{\alpha,1}t}{}_1\langle 1|\hat{u}_{\alpha,1}(t)|1\rangle_1|\alpha\rangle_1) \\
& ({}_2\langle 0|\hat{u}_{\mathrm{vac},2}(t)|1\rangle_2|\mathrm{vac}\rangle_2 + \sum_\alpha e^{-i\omega_{\alpha,2}t}{}_2\langle 0|\hat{u}_{\alpha,2}(t)|1\rangle_2|\alpha\rangle_2) \qquad (4c) \\
|\widetilde{\chi_{1,1}(t)}\rangle = {} & \alpha({}_1\langle 1|\hat{u}_{\mathrm{vac},1}(t)|0\rangle_1|\mathrm{vac}\rangle_1 + \sum_\alpha e^{-i\omega_{\alpha,1}t}{}_1\langle 1|\hat{u}_{\alpha,1}(t)|0\rangle_1|\alpha\rangle_1) \\
& ({}_2\langle 1|\hat{u}_{\mathrm{vac},2}(t)|0\rangle_2|\mathrm{vac}\rangle_2 + \sum_\alpha e^{-i\omega_{\alpha,2}t}{}_2\langle 1|\hat{u}_{\alpha,2}(t)|0\rangle_2|\alpha\rangle_2) \\
& +\beta({}_1\langle 1|\hat{u}_{\mathrm{vac},1}(t)|1\rangle_1|\mathrm{vac}\rangle_1 + \sum_\alpha e^{-i\omega_{\alpha,1}t}{}_1\langle 1|\hat{u}_{\alpha,1}(t)|1\rangle_1|\alpha\rangle_1) \\
& ({}_2\langle 1|\hat{u}_{\mathrm{vac},2}(t)|1\rangle_2|\mathrm{vac}\rangle_2 + \sum_\alpha e^{-i\omega_{\alpha,2}t}{}_2\langle 1|\hat{u}_{\alpha,2}(t)|1\rangle_2|\alpha\rangle_2) \qquad (4d)
\end{aligned}
$$

For the given $|\Psi(t)\rangle$, the density operator $\rho$ of the whole system is the defined as $\rho = |\Psi(t)\rangle\langle\Psi(t)|$. The matrix elements of density matrix of the system takes the following form:

$$
\begin{aligned}
\Lambda_{11} =& \alpha^2\langle 0|\hat{u}_{\text{vac},1}(t)|0\rangle^2\langle 0|\hat{u}_{\text{vac},2}(t)|0\rangle^2 \\
&+\beta^2\langle 0|\hat{u}_{\alpha,1}(t)|1\rangle^2\langle 0|\hat{u}_{\alpha,2}(t)|1\rangle^2 \\
\Lambda_{14} =& \alpha\beta^*\langle 0|\hat{u}_{\text{vac},1}(t)|0\rangle\langle 1|\hat{u}_{\text{vac},1}(t)|1\rangle^*\langle 0|\hat{u}_{\text{vac},2}(t)|0\rangle\langle 1|\hat{u}_{\text{vac},2}(t)|1\rangle^* \\
&+\beta\alpha^*\langle 0|\hat{u}_{\alpha,1}(t)|1\rangle\langle 1|\hat{u}_{\alpha,1}(t)|0\rangle^*\langle 0|\hat{u}_{\alpha,2}(t)|1\rangle\langle 1|\hat{u}_{\alpha,2}(t)|0\rangle^* \\
\Lambda_{22} =& \alpha^2\langle 0|\hat{u}_{\text{vac},1}(t)|0\rangle^2\langle 1|\hat{u}_{\alpha,2}(t)|0\rangle^2 \\
&+\beta^2\langle 0|\hat{u}_{\alpha,1}(t)|1\rangle^2\langle 1|\hat{u}_{\text{vac},2}(t)|1\rangle^2 \\
\Lambda_{23} =& e^{+i\Delta_1 t}e^{-i\Delta_2 t}[\alpha\beta^*\langle 0|\hat{u}_{\text{vac},1}(t)|0\rangle\langle 1|\hat{u}_{\text{vac},1}(t)|1\rangle^*\langle 1|\hat{u}_{\alpha,2}(t)|0\rangle\langle 0|\hat{u}_{\alpha,2}(t)|1\rangle^* \\
&+\beta\alpha^*\langle 0|\hat{u}_{\alpha,1}(t)|1\rangle\langle 1|\hat{u}_{\alpha,1}(t)|0\rangle^*\langle 1|\hat{u}_{\text{vac},2}(t)|1\rangle\langle 0|\hat{u}_{\text{vac},2}(t)|0\rangle^*] \\
\Lambda_{32} =& e^{-i\Delta_1 t}e^{+i\Delta_2 t}[\alpha\beta^*\langle 0|\hat{u}_{\alpha,1}(t)|1\rangle^*\langle 1|\hat{u}_{\alpha,1}(t)|0\rangle\langle 1|\hat{u}_{\text{vac},2}(t)|1\rangle^*\langle 0|\hat{u}_{\text{vac},2}(t)|0\rangle \\
&+\beta\alpha^*\langle 0|\hat{u}_{\text{vac},1}(t)|0\rangle^*\langle 1|\hat{u}_{\text{vac},1}(t)|1\rangle\langle 1|\hat{u}_{\alpha,2}(t)|0\rangle^*\langle 0|\hat{u}_{\alpha,2}(t)|1\rangle] \\
\Lambda_{33} =& \alpha^2\langle 1|\hat{u}_{\alpha,1}(t)|0\rangle^2\langle 0|\hat{u}_{\text{vac},2}(t)|0\rangle^2 \\
&+\beta^2\langle 1|\hat{u}_{\text{vac},1}(t)|1\rangle^2\langle 0|\hat{u}_{\alpha,2}(t)|1\rangle^2 \\
\Lambda_{41} =& \alpha\beta^*\langle 0|\hat{u}_{\alpha,1}(t)|1\rangle^*\langle 1|\hat{u}_{\alpha,1}(t)|0\rangle\langle 0|\hat{u}_{\alpha,2}(t)|1\rangle^*\langle 1|\hat{u}_{\alpha,2}(t)|0\rangle \\
&+\beta\alpha^*\langle 0|\hat{u}_{\text{vac},1}(t)|0\rangle^*\langle 1|\hat{u}_{\text{vac},1}(t)|1\rangle\langle 0|\hat{u}_{\text{vac},2}(t)|0\rangle^*\langle 1|\hat{u}_{\text{vac},2}(t)|1\rangle \\
\Lambda_{44} =& \alpha^2\langle 1|\hat{u}_{\alpha,1}(t)|0\rangle^2\langle 1|\hat{u}_{\alpha,2}(t)|0\rangle^2 \\
&+\beta^2\langle 1|\hat{u}_{\text{vac},1}(t)|1\rangle^2\langle 1|\hat{u}_{\text{vac},2}(t)|1\rangle^2
\end{aligned} \tag{4e}
$$

Exact values used in plotting Fig 2: The dynamics of the probability for initial entangled state of bipartite system of DNA and mRNA, (a) plot of $P(\Psi = \Psi(0))$ as a function of $t$ with the values tunneling strength $\Delta_1 = \Delta_2 = \Delta = 1$ and $\Gamma_1 = \Gamma_2$ are equal to $0.005\Delta$, $0.015\Delta$ and $0.05\Delta$ for *blue*, *red* and *green* curves, respectively, (b) same plot as (a) with the values $\Delta_1 = \Delta_2 = 1$, $\Gamma_1 = 0.05\Delta_1$ and $\Gamma_2 = 0.05\Delta_2$ for *red* curve and $\Delta_1 = 1$, $\Delta_2 = 0.5$, $\Gamma_1 = 0.05\Delta_1$ and $\Gamma_2 = 0.05\Delta_2$ for *blue* curve, (c) same plot as (b) but with the values $\Delta_1 = 1$ and $\Delta_2 = 0.01$ for *blue* curve.

Exact values used in plotting Fig 3: The time-evolution of the concurrence associated to the mRNA-DNA entangled state, (a) plot of $C(\rho)$ as a function of $t$ (dimensionless) with the values $\alpha = \cos\pi/4$, $\beta = \sin\pi/4$, $\Delta_1 = \Delta_2 = 1$, $\Gamma_1 = \Gamma_2$ and also $\Gamma_1$ ($\Gamma_2$) is equal to $\kappa\Delta_1$ ($\kappa\Delta_2$), (b) same as (a) but $\Delta_1 = 1$ and $\Delta_2 = 0.5$, (c) same as (a) but $\Delta_1 = 1$ and $\Delta_2 = 0.01$, (d) 3D plot of $C(\rho)$ as a function of $t$ and $\Delta_2$ with same values as (a) but $\Delta_2$ changes from 0 to 1.

Exact values used in plotting Fig 4: The time-evolution of the entanglement of formation associated to the bipartite mRNA-DNA entangled state $\Psi$ defined in Eq (28) in main text, (a) plot of $E(C)$ as a function of $t$ with the values $\alpha = \cos\pi/4$, $\beta = \sin\pi/4$, $\Delta_1 = \Delta_2 = 1$, $\Gamma_1 = \Gamma_2$ and also $\Gamma_1$ ($\Gamma_2$) is equal to $\kappa\Delta_1$ ($\kappa\Delta_2$) where $\kappa$ takes the values 0.005, 0.01, 0.02 and 0.05 for blue, orange, green and red curves, respectively, (b) same as (a) but $\Delta_1 = 1$ and $\Delta_2 = 0.5$, (c) same as (a) but $\Delta_1 = 1$ and $\Delta_2 = 0.01$.
